# Developing stakeholder participation to address lack of safe water as a community health concern in a rural province in South Africa

**DOI:** 10.1080/16549716.2021.1973715

**Published:** 2021-09-20

**Authors:** Jennifer Hove, Lucia D’Ambruoso, Rhian Twine, Denny Mabetha, Maria van der Merwe, Ishmael Mtungwa, Sonto Khoza, Kathleen Kahn, Sophie Witter

**Affiliations:** a MRC/Wits Rural Public Health and Health Transitions Research Unit (Agincourt), School of Public Health, Faculty of Health Sciences, University of the Witwatersrand, Johannesburg, South Africa; b Aberdeen Centre for Health Data Science (Achds) Institute of Applied Health Sciences, School of Medicine, Medical Sciences and Nutrition, University of Aberdeen, Scotland, UK; c Department of Epidemiology and Global Health, Umeå University, Umeå, Sweden; d National Health Service (NHS) Grampian, Scotland, UK; e Independent Consultant, South Africa; f Department of Health, Mpumalanga Provincial Government, Mbombela, South Africa; g International Network for the Demographic Evaluation of Populations and Their Health (Indepth), Accra, Ghana; h Institute for Global Health and Development, Queen Margaret University, Scotland, UK

**Keywords:** Participatory water governance, multisectoral stakeholders, community evidence, local action plan, water challenges

## Abstract

**Background:**

Despite legislative and policy commitments to participatory water governance in South Africa, and some remarkable achievements, there has been limited progress to improve the water infrastructure servicing in marginalized rural communities. Around five million South Africans still do not have access to safe water.

**Objective:**

This paper seeks to understand and advance processes to engage multisectoral stakeholders to respond to lack of safe water as a community-nominated health priority in rural South Africa.

**Method:**

We engaged representatives from Mpumalanga Department of Health (MDoH), rural communities, other government departments and non-governmental organisations (NGOs) to cooperatively generate, interpret and act on evidence addressing community-nominated priorities. A series of participatory workshops were conducted where stakeholders worked together as co-researchers to develop shared accounts of the problem, and recommendations to address it. Consensus on the problem, mapping existing planning and policy landscapes, and initiating constructive dialogue was facilitated through group discussions in a collective learning process.

**Results:**

Community stakeholders nominated lack of safe water as a local priority public health issue and generated evidence on causes and contributors, and health and social impacts. Together with government and NGO stakeholders, this evidence was corroborated. Stakeholders developed a local action plan through consensus and feasibility appraisal. Actions committed to behavioural change and reorganization of existing services, were relevant to the needs of the local community and were developed with consideration of current policies and strategies. A positive, collective reflection was made on the process. The greatest gain reported was the development of dialogue in ‘safe spaces’ through which mutual understanding, insights into the functioning of other sectors and learning by doing were achieved.

**Conclusion:**

Our process reflected willingness and commitment among stakeholders to work together collectively addressing local water challenges. Location in an established public health observatory helped to create neutral, mediated spaces for participation.

## Background

Diseases related to lack of water, sanitation and hygiene pose significant risks to health, and higher mortality and morbidity in marginalized communities globally [1–3]. In South Africa, the leading causes of under-5 mortality and morbidity are pneumonia and diarrhoea [4–6]. It is a public health concern that many under-5 deaths could be prevented through improved access to clean water [5,7]. Increasing access to clean water and sanitation services are essential in preventing and protecting health in normal times and during disease outbreaks, including the current COVID-19 pandemic, which has further amplified the impacts of water inequities [8].

Access to safe water is recognized as a fundamental right and is enshrined in the South African constitution [9]. While remarkable progress has been made, with approximately 90% of South Africans having access to piped water in 2016 [10], more than two decades since the end of apartheid, rural areas do not have continuous supply [11]. There are substantial differences in water access across provinces, with rural provinces lagging behind [10,12]. From 2002 to 2019, access to water declined in five provinces with the largest decline in Mpumalanga [12]. Today, around 5 million South Africans do not have reliable access to clean drinking water, connected to apartheid legacy, poor public sector accountability and low-average annual rainfall [10,13]. Due to climate change, South Africa faces extreme weather conditions with increasing frequency of drought, heat waves and floods [14]. In addition, poor performance of municipalities has also given rise to increase in service delivery protests, which are often violent and destructive [15–17].

In South Africa, participatory water governance has been embraced as a key enabling mechanism supporting water resource management. The South African 1997 Water Service Act (WSA) and National Water Act (NWA) of 1998 were framed around the concept of Integrated Water Resource Management (IWRM) [9,18,19]. While there is legislation and strong support in theory, policy and principle for community and stakeholder participation in water resource management, engagement has not been effective [20]. Lack of effective participatory water governance remains a challenge, particularly the involvement of stakeholders in priority setting, planning, decision-making and implementation.

The South African government also supports stakeholder participation in health, as an underpinning principle of primary health care (PHC) enshrined in the Alma Ata Declaration of PHC in 1978 [21–24]. Stakeholder’s participation is furthermore key to recent shifts towards National Health Insurance and revival of the district health system, with PHC re-engineering and Ward-Based Primary Health Care Outreach Teams (WBPHCOTs) as key service delivery mechanisms bringing services closer to people [25,26]. There is recognition that decision-making should be a collaborative and inclusive process to address gaps in health system delivery. However, health policy and strategy has not yielded intended results in equity, sustainability and efficiency in health service delivery [23,27].

Understanding of the theoretical benefits of stakeholder participation exist, however, getting it right in practice is the challenge [11,18,28]. There is widespread normative support for stakeholder participation, but there remains limited understanding of how to operationalise the concept in practice. This paper seeks to understand and advance a process of multisectoral stakeholder participation in response to lack of safe water as a community-nominated public health priority in a rural sub-district in Mpumalanga, South Africa. The objective was to engage multisectoral stakeholders to build evidence and dialogue to respond to lack of safe water as a community-nominated public health priority.

There is inconsistency in use of the terms, ‘participation’, ‘involvement’ and ‘engagement’, further complicated by how those involved are named e.g. communities, service users, providers, decision-makers, and researchers. In this paper, we use the term ‘participation’, and refer to community members, service providers, professionals, and researchers as ‘stakeholders’. The premise is that real expertise lies among those for whom health priorities are most directly relevant, hence we adopted the definition of stakeholder participation to include community stakeholders as equals.

## Methods

### Study setting

The study was progressed in Mpumalanga, a rural province with high unemployment (31%) and low economic activity: approximately 23% of households have no regular source of income [29]. While health service delivery has improved since 1994, the lack of income leads to exclusion from access [30]. The study area was the Agincourt (HDSS), located in Bushbuckridge local municipality [30]. The Agincourt HDSS was established in 1992 to better understand population health in rapidly transitioning societies [30]. The HDSS consists of 120,000 people living in 31 villages in 21,500 households [30]. Xitsonga Tsonga is the most commonly spoken language in the area [30]. The Agincourt HDSS provides data for health population planning for the district from marginalized communities. The HDSS, a stable public health observatory, enabled the development of a dialogue process with different levels and sections in the health systems, adjacent government departments, NGOs and rural communities. The study was part of a wider programme, Verbal Autopsy with Participatory Action Research (VAPAR) (www.vapar.org), which aims to expand the knowledge base through partnership for action on health equity [31]. Verbal autopsy (VA) is a method to understand levels and causes of deaths in otherwise unregistered populations. Participatory Action Research (PAR) is a non-linear community-based process that systemizes local evidence for action with iterative learning cycles following a stepwise approach [32]. The study protocol was developed together with co-researchers based on pilot work in 2015–2018 [33–35].

### Generating community knowledge and combining with routine mortality data

We engaged community stakeholders to work as co-researchers to generate evidence on local priorities. We progressed a PAR process with community stakeholders in three villages in the Agincourt HDSS who had participated in the pilot phase of the VAPAR programme from 2015 to 2016 (Table 1) [34]. In the pilot work, participants were purposively recruited to understand the subjective reality and experiences of health using a maximum variation sampling technique. Participants represented a cross-section of the community including community and religious leaders, community health workers, clinic committee members, traditional healers and family members. The research team telephonically contacted participants from the pilot phase for recruitment, described the study, activities and intended outputs.

**Table 1.** Composition of workshop

Participants**	Generating community evidence*	Analyzing and interpreting community evidence			Collectively planning local action	
	Workshops					
	1–8	1	2	3	1	2
Religious leader	1	-	-			
Traditional leader	3	-	-			
Community official	5	-	-	1	1	1
Community Health Worker	3	-	-	1	1	
Family member	11	-	-	1	1	
Women of reproductive age	9	-	-			
Youth	16	-	-			1
Department of Health- Province		12	6	2	4	2
Department of Health- District		1	3	3	5	5
Department of Cooperative Governance and Traditional Affairs		-	1	1	-	1
Department of Social Development - Province		-	1	2	2	1
Department of Home Affairs		-	1	-	-	-
Department of Basic Education		-	1	1	-	-
Department of Water and Sanitation		-	-	2	1	-
Department of Culture, Sports and Recreation		-	-	1	1	-
Inkomati Usuthu Catchment Management Agency (IUCMA)		-	-	-	-	1
Ward counsellor		-	-	-	-	1
Ward committee member		-	-	-	-	1
Africa Foundation		-	-	-	-	1
Researchers	4	5	4	3	3	5
Total	52***	18	17	18	19	20

*Results presented elsewhere [36,37]. All participants were 18 years or older. Participants** were acknowledged as having multiple roles at home and in the community and a primary role was identified with participants for the purposes of recruitment. The total of 52*** include 32 participants from the other 2 villages that nominated a different priority, reported elsewhere [36].

During this phase, eight workshops of 2–3 hours each were held once a week from June to September 2017. In each village, an initial workshop was held with eight participants, then the study was introduced, and the process was co-designed. Photovoice was also used to capture lived realities of water insecurity. All eight participants were provided with digital cameras and were trained how to use them. ‘Lack of safe water’ was nominated by community stakeholders in one village as a local priority health concern. Participants identified women of reproductive age as people affected by, and with knowledge on lack of safe water, and eight additional participants were recruited to bring in perspective otherwise excluded from the village that nominated water.

A further seven workshops were then held with 16 community stakeholders using PAR methods to develop and verify a collective understanding of the problem and actions to address it. PAR principles guided the workshops, i.e. homogeneous group (shared condition), subjective perspective (individual experiences), collective validation (issues recognized by the group as important) and no delegation (those dealing with the issue are primary researchers). Additionally, VA data from the Agincourt HDSS were collated and presented to demonstrate the health impacts of lack of water in communities. Workshop facilitation was a shared responsibility between the VAPAR research team and community participants, increasingly shifting to community led discussions over a series of engagements. All the workshop outputs and transcripts were back translated for quality control. Results from this stage, published elsewhere [36,37], were organised into a research brief, which was subsequently used in engaging government and NGO stakeholders.

### Analysing and interpreting community evidence with multisectoral stakeholders

We then held three multisectoral workshops to analyse and interpret the VA and PAR data with community, government and NGO stakeholders from January to March 2018. The first workshop convened MDoH officials from the provincial and district structures at MDoH offices in the city of Mbombela (Table 1). The research brief was discussed, and PAR techniques: group model building, and rich pictures, were used to build consensus around and shared accounts of the issue. Group model building engages different stakeholders in a process for developing shared understanding and addressing complex problems [38]. Rich pictures are detailed, visual hand-drawn representations of discussions used as a tool for learning about complex, ill-defined problems [39]. Stakeholders also considered in detail the regulatory, policy and programmatic contexts in which interventions could be introduced.

The second and third analysis workshops were co-designed in the first workshop. This included identification of stakeholders, logistics and recruitment processes. In the second analysis workshop, recommendations for action were collectively developed, with a focus on local implementation contexts. The final workshop included representatives from various sectors and community stakeholders. Stakeholders adopted active roles in developing and presenting the group model building, rich pictures, and action recommendations. Open discussion was facilitated, which ratified recommendations and identified challenges, opportunities, and strategies to address lack of safe water, within local, operational contexts through a consensus-building process.

### Collectively planning local action

In the final stage, two planning workshops were held at district level in Agincourt HDSS field offices to develop a local action plan from September to October 2018. Attendees comprised district, sub-district and local MDoH stakeholders, and adjacent governmental department district level stakeholders (Table 1). In the first workshop the topic was revised to accommodate priorities of both community and MDoH stakeholders; lack of safe water and its effect on child health. Facilitated discussions were held to identify and appraise actions. For each action, actors, feasibility, specific change expected, completion date, resources available or needed and communication channels were discussed, agreed and noted. Local implementation contexts were also mapped. Complex inter-relationships were mapped, formal and informal spheres of action identified, and potential leverage points to influence systems change were identified.

In all workshops, stakeholders collectively analysed evidence and progressed reflective dialogue to reach consensus, reviewing workshop outputs to ascertain that overarching questions were answered. At the end of each step, collective reflections were made, and reports generated and shared. All workshops were discursive, with facilitators ensuring that discussions were focused, substantive, inclusive and respectful, using facilitation guides.

### Data management and analysis

**Figure 1.**
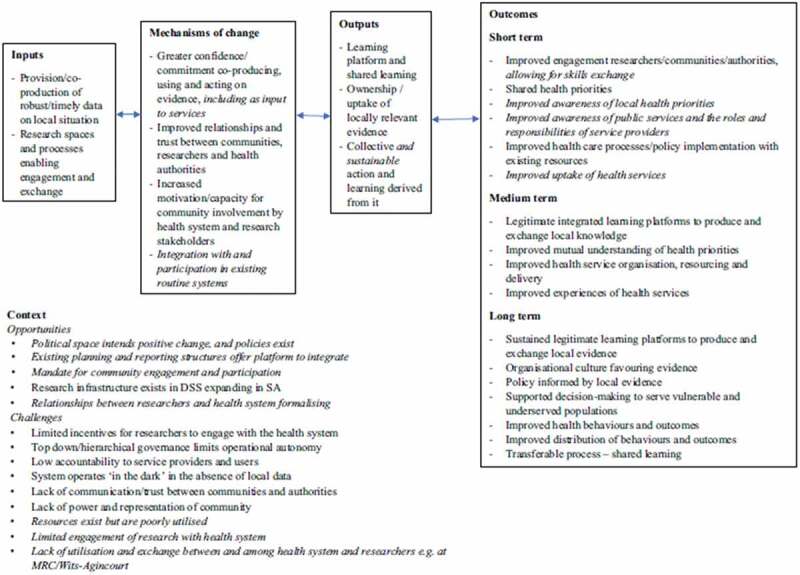
VAPAR Programme theory of change; *Source* [31,40]

Data comprised the workshop outputs, including group models, rich pictures, reports, observational notes, attendance registers and researchers’ reflective journals. We used thematic analysis to develop understandings of how evidence is created with reference to stakeholder perspectives, how the process could be used to influence provincial and district-level planning, and to develop shared agendas, action plans and partnerships. Analysis was guided by the overall VAPAR theory of change (ToC) developed based on continuous engagement with multiple stakeholders [31,40] to explore the context, role of evidence, mechanisms that lead to change, actors and how they learn from each other (Figure 1).Community evidence was co-produced, stakeholders shared knowledge on their own situations, and dialogue was built and collective action developed, reflecting  on and learning the process with the authorities. Data familiarization and generation of initial codes was performed by DM, RT, LD and JH to verify whether the content reflected the most important concepts. Codes were then grouped into themes using combined inductive (emerging themes) and deductive (ToC) generation until thematic saturation. Data was stored on secure servers at the University of Aberdeen and Agincourt HDSS.

## Results

### Community knowledge and routine mortality data

In the PAR element, community stakeholders developed sophisticated, multi-level accounts of the local water situation, narrating repeated, and prolonged periods without piped water, surface water sources (such as rivers and dams), unreliable infrastructure, inadequate service delivery, empty reservoirs and various health and social impacts. Stakeholders overwhelmingly attributed lack of safe water to poor planning by water authorities and service providers. Community stakeholders collectively developed potential strategies and solutions to address lack of clean water. Analysis of VA data revealed that approximately 34% of the deaths recorded in the Agincourt HDSS (2014/15) could reasonably be attributed to lack of clean water. A research brief presenting the VA and PAR data was developed.

### Analyzing data with multisectoral stakeholders

**Figure 2.**
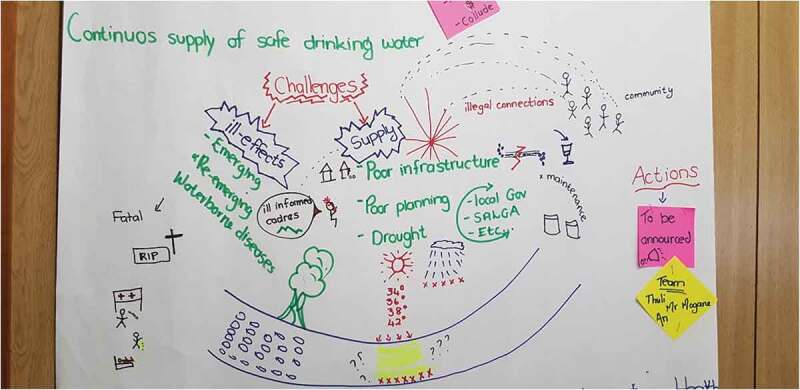
Rich picture exploring barriers to lack of safe water in rural communities

In the subsequent multisectoral stakeholder workshops, government actors validated the community and statistical evidence on causes and contributors, health, and social impacts of lack of safe water and potential actions to address water shortages (Table 2). MDoH stakeholders developed consensus with and verified community stakeholders’ accounts that lack of clean water was a result of complex barriers faced by water services providers and authorities. Rich situated accounts of system, and structural drivers of the problem were developed throughout the process (Figure 2).

**Table 2.** Barriers, solutions and existing policy related to lack of safe water developed by stakeholders analyzing evidence from the community

Issue	Activities
Problem definition:Barriers	Illegal water connections, vandalism of infrastructure Poor infrastructure- lack of maintenance Poor planning and financial mismanagement Incompetency of local Government officials/ high staff turnover Outsourcing – inflate cost and potential to collude. Contamination of catchment areas/deterioration in wastewater treatment Lack of data and information on water management due to lack of monitoring system Drought Policy fragmentation
Solutions	Community participation to facilitate ownership and accountability, reporting of water leakages and vandalism. Health education at household level through Ward-Based Primary Health Care Outreach Teams (WBPHCOTs), community health workers, ward committee and community meetings, at facility by clinicians and at school-by-school heath teams. Provide water infrastructure and maintenance of existing infrastructure. Capacity building of officials Monitor implementations of plans Supervision and mentoring Life skills orientation Integration of services Awareness campaign through community mass meetings
Existing Policies	National Water Resource strategy (NWRS) aims to improve water demand management, water conservation and capacity building and skills development. Water and sanitation forums Capacity building among communities and their municipality leaders 2020 vision for water and sanitation education programme Councilor development program with South Africa Local Government Association (SALGA) and, Cooperative Governance and Traditional Affairs (COGTA) To educate and empower community councilors about water related issues. Integrated Development Plan (IDP) A 5-year plan that prioritizes community needs by allowing members to identify need and be part of the implementation plan. It has bulk municipal infrastructure grants and plays as a link for community engagement
**Insights and recommendations** Municipal legislation: Municipalities to develop bylaws and provide law enforcers to deal with criminal acts such as illegal connections and vandalism. Revenue: The funding for infrastructure maintenance should be adequate. Secondly Bushbuckridge municipality relies on government grants, and this need be to be addressed for municipalities to sustainably function. Municipality officials should account for the use of funds and their activities to the community. Water reading meters should be installed to improve revenue collection. Community awareness and education: Communal standpipes did not have continuous supply, communities need to know their water rights, take ownership, and be involved in decision making. Need to protect water infrastructure. Education perhaps may empower the community to shift their mindset towards the perception that government services should be provided free of charge. Integration approach: There is need for integration of policies and collaboration across various government departments. Participants mentioned the need to review the Integrated Development Plan (IDP) annually because at times priorities are overwritten by emergent issues that may have to be addressed immediately. Policies should be understood, information collated, and managed and MRC/Wits Agincourt monitored diligently.

Stakeholders agreed that municipalities are crippled with lack of sufficient resources to sustain activities, despite their willingness to provide water services to the people. Discussions revealed that municipalities generate revenue through rates and taxes, but the Bushbuckridge municipality mostly relied on conditional government grants and cannot generate their own revenue. This is due to it being deeply rural and a former homeland area with no rates collection system, unlike urban municipalities.

Stakeholders also acknowledged the complexity of water resource management, including lack of household water usage monitoring, resulting in lack of power to control water levels, monitor usage and leakage and recover cost. Low accountability of water authorities to service providers and users, and lack of communication with communities were identified as further contextual challenges outlined in the ToC, which the VAPAR programme seeks to address. The situation was verified, and arguably worsened, by frequent violent destructive service delivery protests, seen to be on the rise. Equitable collaboration which our process facilitated, enhanced the feeling of joint ownership, buy in and acceptability and stakeholders recognized the importance of meaningful community dialogue to build mutual understanding and trust instead of protesting.

The workshop discussions focused in detail on accountability while it was agreed that water is a social determinant of health, MDoH stakeholders reported no direct statutory mandate to be involved in addressing the issue. However, MDoH stakeholders expressed obligations to respect and honour community priorities by engaging in multisectoral learning and developing action with adjacent departments. MDoH stakeholders acknowledged that the actions to address these were required at different levels in the health system, and in collaboration with others. They also repeatedly acknowledged the benefits of understanding the roles, functions and policies of other departments, as they pertained to health protection and disease prevention.

As part of understanding the local policy and strategy contexts, two main policies related to access to safe water were discussed with multisectoral stakeholders. First, the NWA of 1998 was described as providing guidelines on how South Africa manages water resources, copes with climate change, and plans for the growing population. Second, the WSA was identified which regulates municipal water supply and sanitation services (Appendix 1). A strong policy context notwithstanding, stakeholders further identified ‘policy noise’ as a barrier to effective service delivery, with policies, strategies and programmes frequently revised before being fully implemented.

**Figure 3.**
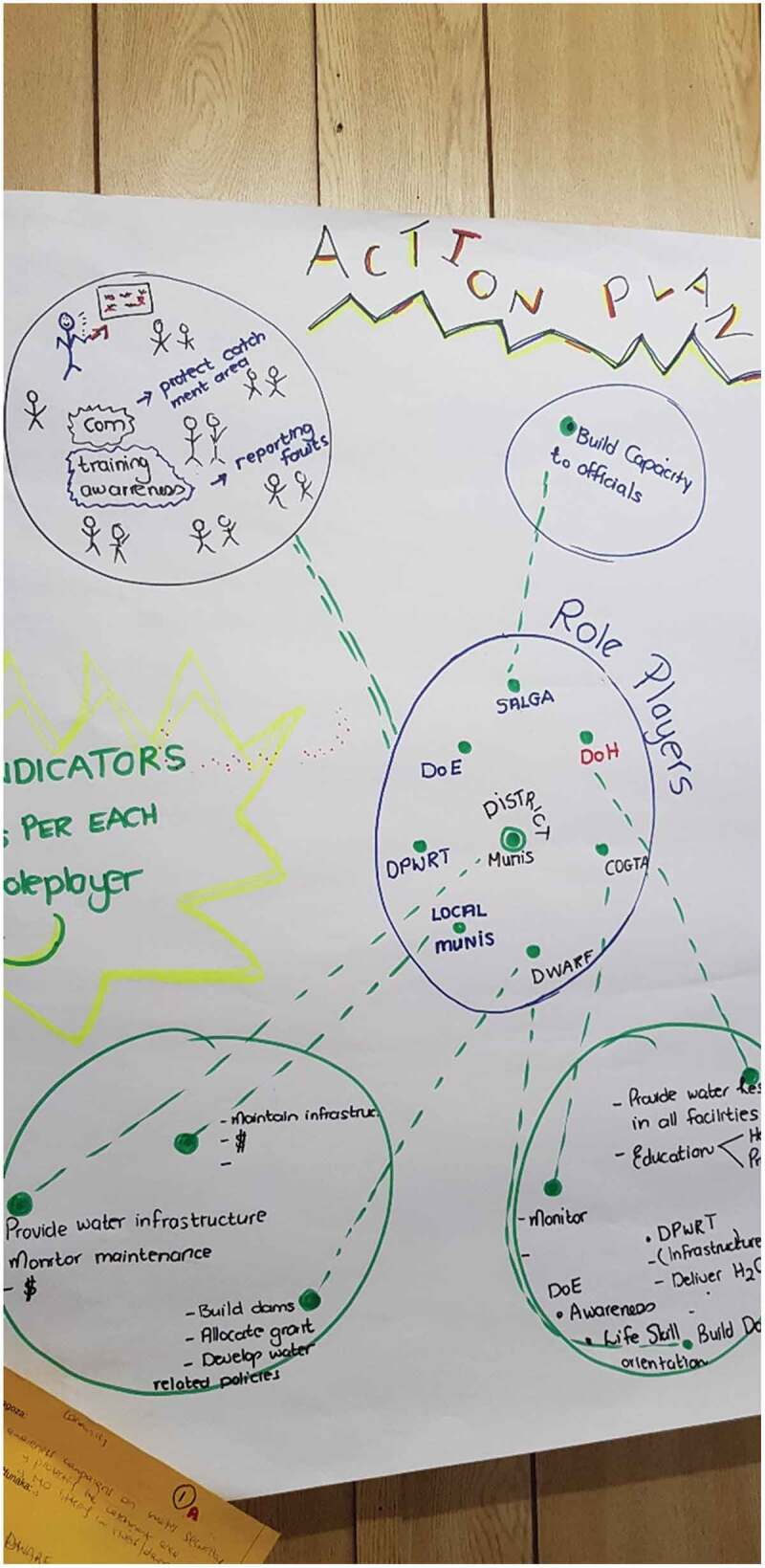
Action recommendations

The National Water Resource Strategy (NWRS), introduced in 2004 through the NWA of 1998, requires the Minister to deliver a National Water Resources Strategy (NWRS) plan every 5 years, providing information on how much water is available in the country and how much is utilised. Stakeholders were concerned that the information provided by the NWRS is not reliable as it does not consider all sectors, though it intends positive changes. In developing recommendations for action, stakeholders focused on lack of multisectoral integration of policy and implementation. These deliberations highlighted lack of community dialogue and trusting relationships that are currently lacking and which are needed to improve relationships between communities, researchers, and the authorities (Figure 3).

A significant policy implementation divide was thus identified and substantiated by stakeholders’ analysis of community and statistical evidence, with recourse to policy, strategies and tactics, and lived experience of implementation among government officials. A recommendation was made for urgent collaboration between sectors to reduce duplication of effort and increase multi-agency cooperation. Stakeholders reiterated that water is a shared resource, hence decisions for water management are made by multiple parties. However, it was agreed that this requires leaders who are committed to create and facilitate partnerships and that the current leadership in the different water management institutions lack shared vision, and that this results in lack of coordination of the multiple perspectives.

### Planning local action

The process culminated in a series of action items, collectively developed by stakeholders, and appraised for feasibility, and recorded in a local action plan to address lack of safe water (Table 3). Based on the realization that complex challenges in water management requires effective, joint leadership from various government stakeholders, stakeholders agreed on a series of shared local actions. Different perspectives were aligned through assessing key dimensions; financial, human resources, time, and sustainability. The action items which were developed, and committed to, involved a range of commitments from immediate low-cost or no-cost behaviour change, to those related to re-organisation of existing services. Through the deliberative process, the local action plan was constructed with reference to, and embedded within, the local context, current key policies and strategies.

**Table 3.** Local action plan

Action	Actors	Measure	Baseline	Target
**Community level**				
Develop clean-up campaigns	Led by community stakeholders with support from: MTPA, Africa Foundation, EHP, DARDLEA and IUCMA	Partnerships with potential stakeholders developed	Number of clean up campaigns done	Involve all potential stakeholders in two IUCMA cleaning campaigns
Advocate for recycling centres	Community, Game Reserves, EHP, DARDLEA	Campaigns hosted	Nothing	Engagement with possible stakeholders on establishment of recycling stations in the community
	SANPARKs – Kruger			
	IUCMA, SSW, MPTA			
**Government level**				
Improve water harvesting	Led by DoBE via schools, DoH – school health nurses, Africa Foundation	Clear understanding of the feasibility of water harvesting	Nothing – some schools had it but infrastructure not maintained	Increased awareness on water harvesting at schools and clinics
Amplify IUCMA awareness campaigns	Led by IUCMA, supported Municipal health services, EPH, Rand Water, WBPHCOTs, ward councillors	Four awareness campaigns supported in BBR by IUCMA and four by SSW	Events have been happening already but not including DoH, DSD and other stakeholders	Stakeholders around water use
Rebuild trust between communities and municipality	Municipality, Ward Councillors, and researchers	Waiting period for municipality to respond to community’s need and community reports on vandalism and other water related challenges	Nothing	Activism campaign – we as South Africans coming together: ‘LET’S TALK ABOUT IT’ to enable political tolerance.
Test water quality- water storage tanks, boreholes, and tankers	Rand Water (BBR Water Board) to work in collaboration with private/NGO partners	Clarity on roles and responsibilities on testing water	Some water testing currently being done by DoH, EHPs at some clinics	Group of potential role-players identified.
				Discussions initiated with municipality
Raise awareness of VAPAR with BBR Executive Mayor	Led by Wits, Ward Councillor and VAPAR team members	Project information and findings shared with Exec Mayor. Records of shared information, activities and dialogue	Nothing	Information report given with our evidence and some recommendations

MTPA; Mpumalanga Tourism and Parks Agency, EHP; Environmental Health Practitioner, DARDLEA; Department of Agriculture Development, Land and Environmental Affairs, IUCMA; Inkomati-Usuthu Catchment Management Agency, SANPARKS; South African National Parks, SSW; Sibanye Stillwater Ltd, DoBE; Department of Basic Education, BBR; Bushbuckridge.

Community stakeholders committed to lead two items on the local action plan i.e. river clean up campaigns and advocating for a recycling centre. The Department of Basic Education committed to lead an awareness drive for water harvesting in schools and clinics. Various stakeholders committed to amplifying Inkomati-Usuthu Catchment Management Agency (IUCMA) awareness campaigns on changing behaviour and perception on water consumption and lifestyle habits through community programmes and water and sanitation forums. Stakeholders suggested that educating the community and leaders may help address challenges of accountability to a point where communities would report leakages, service delivery breakdowns, vandalism, illegal connections, and possibly shift in mind-sets regarding paying for services.

The communication gap between the municipality and community regarding lack of knowledge on how the municipality functions prompted discussion on trust issues. Rebuilding a trusting relationship between the community and the municipality was discussed as essential. Stakeholders suggested that municipalities should introduce bylaws, strengthen existing laws and punitive measures (enforcers) to deal with illegal water connections, and address the current dependency on social grants through facilitating dialogue and involving the communities in decision making.

Maintenance of infrastructure in schools, clinics and villages was also cited as a mechanism to rebuild trust with communities. In addition, stakeholders cited that the current billing system is obsolete and needs reform, aligning it to the right to water. Municipalities use a flat rate and there are no meters installed on properties to measure actual water consumption. Moreover, revenue is only collected from schools, businesses and hospitals, and residents in the study area get water free of charge which is different from urban or metro municipality.

The municipality and Bushbuckridge Water Board led an action to initiate and advocate for collaboration between private and NGO partners to test for water quality. The final item on the local action plan was for researchers to raise awareness of the VAPAR programme with the Bushbuckridge mayor, to engage, build networks and make evidence accessible to policy makers. This work is part of the wider VAPAR process that involves progressing, monitoring, and reflecting on and learning from the process of implementing the action plan. The VAPAR programme progressed a process of implementing and evaluating the local action plan, which is reported elsewhere [40].

### Collective reflections on the process

Throughout, participants reflected on the process and the programme ToC was revised [31,40]. Overall, stakeholders reflected positively. There was a high level of attendance and active involvement in all five workshops. The VAPAR process created a platform where power imbalances were mitigated. We co-designed a process to build community capabilities to generate and act on knowledge on their situations. This was combined with statistical evidence from Agincourt HDSS as a basis for engagement with multi-stakeholders to develop collective action to respond to lack of safe water. Community stakeholders felt empowered and reported increased confidence to collectively act on evidence leading to implementation of some of the action items on the local action plan. Involving stakeholders as partners in analyzing and interpreting community evidence brought about changes of attitude and increased ownership by both community and government stakeholders. Community stakeholders reported improved awareness of public services and the roles and responsibilities of service providers. Community stakeholders were initially negative and blaming towards authorities, but with continuous engagement, followed by sensitive facilitated dialogue with the authorities, developed more constructive collaborative mindsets. Group model building, rich pictures and action recommendations facilitated open discussion and built shared understandings of the problem.

Through the process, positionality of the researchers was a critical component. Choices on how the process should progress was negotiated by researchers and stakeholders. For example, dates, time, duration of the meeting, new stakeholder recruitment and inclusion of voices typically excluded were mutually agreed to be suitable and not intrusive or disruptive of other commitments and were specifically designed to share power, and control and enable co-ownership. Collaboration between various stakeholders was also key in developing local action plans, creating learning platforms, and shared learning. MDoH stakeholders stated that the process offered an opportunity to meet with, learn about and link with other stakeholders outside the health system. For some, this was the greatest gain. The process raised awareness on the need for collective planning and action monitoring. Power asymmetries were managed through facilitating the workshops sensitively, creating safe spaces in which we had open and transparent discussion. Some government stakeholders felt that they learnt to respect the opinions of others, including community stakeholders, through shared intellectual constructs and co-developing knowledge.

The biggest challenge mentioned was sustaining continuous stakeholder engagement with busy stakeholders. Participants suggested more time for deliberations, more meetings with affected communities, involvement of more communities, consistent availability of participants and direct involvement of all stakeholders so that actions do not end on paper but are implemented and evaluated to determine their effectiveness and efficacy.

## Discussion

It is now more than two decades since the enactment of the 1997 WSA and 1998 NWA, in which stakeholder participation is enshrined. Despite this, deep challenges of inequities in access to clean water remain in South Africa [10]. Our data suggest limited cross-sectoral dialogue, limited engagement between the authorities and communities, and integration of different departments in the production of strategies and programmes. The local municipality in the Agincourt HDSS struggles to continuously supply safe water; a common phenomenon in rural municipalities across South Africa [41,42]. This finding is consistent with other studies. Rural municipalities predominately face a number of challenges in providing services to the communities as per their constitutional mandate [43]. A study in Makana Local Municipality in the Eastern Province of South Africa identified similar problems to those in Bushbuckridge i.e. high unemployment and poverty rates [10], and poor billing systems resulting in many residents not affording to pay for water services [44]. This hampered the Makana local municipality in supporting staff to operate and maintain infrastructure [44,45]. Our findings are furthermore consistent with other research that lack of safe water has multiple, complex determinants which are systemic and cannot be solved by simple solutions, requiring effective multisectoral collaboration [20,46–49]. Therefore, if authorities work with communities and are responsive to community issues through participatory multi-sectoral collaboration, problems of lack of safe water could be improved, relationships and trust between communities, and researchers and authorities will be restored.

A process of inclusive stakeholder participation confirmed this and allowed us to understand the contextual drivers of lack of safe water through prioritizing local health issues, generating locally relevant evidence and interpreting, analyzing, and planning for action. Local knowledge and statistical data were integrated and formalized the process into co-owned commitments to integrated action. Situating the discussions in neutral spaces reflecting implementation contexts, not formal government spaces, in addressing lack of clean water and developing local action plans was critical not only in amplifying the voice of the community but also in developing mutual understanding and building relationships among different stakeholders who could possibly influence change. Despite water being a social determinant of health, the MDoH had less direct influence, but what was key in our process was building relationships and networks. This reflects the need for multisectoral stakeholder processes, which have the potential to support co-production of knowledge, creating learning platforms and collective action. The results are thus consistent with evidence that suggests effective participation and sustainable outcomes take place when multiple stakeholders, including services users, are part of the process leading to change [40,47,50–52]. It is argued that plans for collaborative action are best developed by people affected by the problem [53,54], and has the potential to inform service delivery and policy [40,49].

Past research has shown that stakeholder participation has not been successful in changing institutions and practices due to the lack of collaboration in designing and planning, resulting in unnecessary policy implementation delays from competing economic, political and social priorities [18,20,50,55–57]. Our process provided a platform for multisectoral collaboration, showing that equitable collaboration between researchers, service providers and service users is possible. The ethics of consensus decision-making was instilled and commitment to equity and transformation was made. Engaging stakeholders from provincial and district health systems with researchers in a learning platform may promote co-production of locally relevant research evidence to address local health priorities. Our findings are consistent with other studies; for knowledge to be used in policies, there is need for it not only to be embedded in relationships, but also to be linked within priorities and contexts of organizations [33,58–62]. Meessen *et al* [63] found that the feasibility of collaborative learning could be hampered by lack of supportive leadership at higher levels [63]. Transforming organizational cultures will be difficult without support at a high level to institutionalize new-learning processes [64,65]. Our process emphasizes the need to consider contextual issues such as accountability, supportive policy, and earlier engagement of diverse stakeholders with a local evidence base.

Our findings are consistent with previous research that though complementary policies and strategies exist within different sectors, multi-sectoral collaboration is surrounded by structural, cultural and cognitive challenges [48,53,63]. However, our process recognize that contributors to lack of access to safe water are multi-faceted, and as such require a cross-sectoral dialogue and integrated approach in planning, designing and implementation. Gaining understanding on the functions and realities of each other’s operations and context is essential for successful collaboration [48,58]. Situated within a stable public health observatory with a long-term presence in rural communities, the VAPAR programme connected different stakeholders from various sectors to tackle lack of safe water at multiple levels and develop local action plans to be implemented in the next step of this research project. As the process continues, the focus will be on sustaining partnerships, transferability of this process to other HDSSs, and evaluation of action.

### Strengths and limitations

Stakeholders co-produced research evidence and created spaces and opportunities for government, and NGO stakeholders to interact with community stakeholders, who experience health inequities deeply. In addition, the process facilitated co-learning across sectors, through doing and reflecting, gaining mutual understanding and knowledge, sharing of information, improved relationships. Building collective capabilities to produce and act on evidence of local relevance was an integral part of the VAPAR programme. For some stakeholders, the process improved stakeholder’s confidence in their problem-solving abilities, and there was increased ownership of the process signified by commitment to act on evidence. The importance of controlling and owning the process was recognized as key to sustainability of programmes and future collaboration.

While involving community stakeholders is key in production of locally relevant evidence, participation in these platforms may be hindered by powerful stakeholders. However, the multi-sectoral stakeholder participation was directed by the community priority, therefore elite capture was less of a concern. Our process within the Agincourt HDSS provided a platform for multisectoral collaboration, without blame, where all stakeholders had equal speaking rights and consensus decision making was encouraged. In addition, sensitive and careful facilitation ensuring inclusive participation and constructive dialogue was helpful. The iterative, adaptative process revealed opportunities to address the topic through development of local action plans. However, how to progress and transfer this process in practice outside the Agincourt HDSS is less straightforward. Maintenance and continuation of participation beyond the project life may not be guaranteed due to the nature of the research. In South Africa, more HDSSs are being established, and they may serve as platforms to roll out this work.

## Conclusions

In this study, bringing diverse stakeholders together was crucial and showed the potential to pool knowledge, experience, expertise and generative collective knowledge and action for locally relevant and acceptable action progressed collectively with various stakeholders. The VAPAR cooperative learning process, based within a stable HDSS setting, provided a conducive environment to produce local research evidence, learn and connect community members, service providers and researchers to tackle challenges in service delivery. When people affected are involved in planning, designing, and developing action, actions are perceived to be socially embedded and culturally acceptable, and may reduce the gap between evidence and practice. On this foundation, the subsequent steps in the process, implementing on and learning from action, will help us to understand how learning and local research evidence can translate into action.

## Data Availability

Data are available on reasonable request to the corresponding author.

## Paper context

Community participation can improve service delivery and health outcomes. Involving multisectoral stakeholders to interpret and analyse participatory action research evidence from the community and develop local action is not only critical in amplifying the voice of the community but also in developing mutual understanding, trust and building relationships among different stakeholders who could possibly influence change. Situating discussions in implementation contexts in addressing lack of safe water were key in facilitating cross sectoral dialogue and integration and engagement of communities, authorities, and researchers.
